# Structural Analysis of Human Cofilin 2/Filamentous Actin Assemblies: Atomic-Resolution Insights from Magic Angle Spinning NMR Spectroscopy

**DOI:** 10.1038/srep44506

**Published:** 2017-03-17

**Authors:** Jenna Yehl, Elena Kudryashova, Emil Reisler, Dmitri Kudryashov, Tatyana Polenova

**Affiliations:** 1 Department of Chemistry and Biochemistry, University of Delaware, Newark, Delaware 19716, USA; 2 Department of Chemistry and Biochemistry, The Ohio State University, Columbus, OH 43210, USA; 3 Department of Chemistry and Biochemistry, University of California, Los Angeles, CA 90095, USA; 4 Molecular Biology Institute, University of California, Los Angeles, CA 90095, USA.

## Abstract

Cellular actin dynamics is an essential element of numerous cellular processes, such as cell motility, cell division and endocytosis. Actin’s involvement in these processes is mediated by many actin-binding proteins, among which the cofilin family plays unique and essential role in accelerating actin treadmilling in filamentous actin (F-actin) in a nucleotide-state dependent manner. Cofilin preferentially interacts with older filaments by recognizing time-dependent changes in F-actin structure associated with the hydrolysis of ATP and release of inorganic phosphate (P_i_) from the nucleotide cleft of actin. The structure of cofilin on F-actin and the details of the intermolecular interface remain poorly understood at atomic resolution. Here we report atomic-level characterization by magic angle spinning (MAS) NMR of the muscle isoform of human cofilin 2 (CFL2) bound to F-actin. We demonstrate that resonance assignments for the majority of atoms are readily accomplished and we derive the intermolecular interface between CFL2 and F-actin. The MAS NMR approach reported here establishes the foundation for atomic-resolution characterization of a broad range of actin-associated proteins bound to F-actin.

Actin is one of the most abundant proteins in eukaryotic cells and is essential for numerous cellular functions, such as cellular division, cell motility, cell shape changes and mechanical support^1^. In these processes, actin functions in a tightly controlled equilibrium between G- (globular) and F- (filamentous) states (Fig. 1a). ATP bound to G-actin is hydrolyzed upon polymerization in a time-dependent manner so that the younger part of the filament contains subunits in the ATP- and ADP-P_i_-states, while the older part is enriched in the ADP-bound subunits (Fig. 1b). This “nucleotide clock” is recognized and amplified by actin-binding proteins (ABPs), among which ADF/cofilins are essential modulators and coordinators of actin dynamics^2^. There are three non-redundant isoforms of human cofilins, namely actin depolymerizing factor (ADF), cofilin 1 (CFL1), and cofilin 2 (CFL2), which differ in tissue distribution, physiological roles, and in the extent they affect actin dynamics. ADF/cofilins bind to ADP-G-actin with 10–50 fold higher affinity than to the ATP-G-actin^3^,^4^,^5^,^6^. A similar tendency is retained upon high-affinity cofilin binding to ADP-state (older filament, higher affinity) as opposed to its inefficient binding to ATP- and ADP-P_i_ states (younger filament, lower affinity) of F-actin^5^,^7^,^8^,^9^. ADF/cofilins regulate actin dynamics by severing actin filaments at the boundaries between bare and coflin-decorated areas under sub-saturating concentrations, while stabilizing F-actin in a new, cofilin-decorated state upon filament saturation^5^,^10^. In vertebrate actins, the severing appears to be mediated by a release of a cation (Mg^2+^ or K^+^ under physiological conditions) from a “stiffness” site between the D-loop and W-loop of two longitudinally adjacent actin subunits^11^. F-actin severing by ADF/cofilins generates new ends and thereby, in coordination with other proteins (e.g. twinfilin, Aip1, CAP), overcomes slow depolymerization as the rate-limiting step in recycling of aged actin filaments.

Budding yeast and human cofilins share ~38% identity and 59% homology, while the three human isoforms of ADF/cofilins share 70–81% identity and 83–89% homology. The structure of ADF/cofilins is highly conserved across all eukaryotic species. The core of ADF/cofilins is comprised of a five-stranded mixed β-sheet and is surrounded by five peripheral α-helices. The secondary structure elements are positioned in the following order: α1-α2-β1-β2-α3-β3-β4-α4-β5-α5-β6. The C-terminus of cofilins is folded in a compact β6-strand, which distinguishes it from a less tightly packed C-terminus of ADF^12^; this difference is translated to a lower affinity of the latter to F-actin. Structural and mutagenesis studies as well as mapping with synchrotron irradiation and chemical cross-linking^12^,^13^,^14^,^15^,^16^ converged to realization that ADF/cofilins interact with actin via two major areas called G/F- and F-binding sites. The former is involved in binding to both monomeric and filamentous actin and is mainly composed of the N-terminal residues, and the kinked α4-helix. The N-terminus contains a major regulatory residue Ser3, whose phosphorylation by LIM kinase^17^ strongly inhibits binding of cofilins to G- and F-actin, as do point mutations at the N-terminus and in the α4-helix^12^,^18^,^19^. The F-actin binding site does not interact with G-actin, but is essential for binding to actin filaments; respectively, mutations in this region (e.g. K96Q^12^) do not affect binding to monomeric actin, but block the ability of cofilin to affect F-actin dynamics. The unique role of ADF/coflins in actin dynamics is directly linked to its ability to induce dramatic structural perturbations in F-actin. Cofilin binding to F-actin promotes relative reorientation of the actin subunits leading to a change in a helical twist of the filament^20^, and an increase in its torsional and bending flexibility^21^,^22^. Cofilin modulates filament interaction with actin binding proteins via direct competition/cooperation, via allosteric influences, or both^23^,^24^,^25^,^26^. Recent cryo-EM reconstruction studies^13^ revealed that reorientation of actin subunits is required to avoid steric clashes between the α1- and α4-helices of cofilin with actin subdomains 1 and 2 (SD1 and SD2), respectively. Thus, binding of cofilin causes a rotation of the outer domain of actin (comprised of SD1, 2) and weakens the interface between SD1 and SD2 of the longitudinally adjacent actin subunits. While these studies yielded key insights, advanced understanding of cofilin-actin interaction and the molecular mechanism of cofilin-based actin remodeling requires atomic level structural information on these complexes, which is currently lacking.

In this report, we present atomic-resolution structural analysis of the muscle isoform of human cofilin (CFL2), bound to the α-skeletal isoform of mammalian F-actin, by magic angle spinning (MAS) NMR spectroscopy. The three isoforms of mammalian cofilins are indispensable and vary in distribution in normal and diseased tissues^3^,^27^,^28^, abilities to support cellular activities^29^,^30^, and in their affinities for G- and F-actin^3^,^9^,^31^,^32^. Among these, only CFL2, the muscle-specific isoform of ADF/cofilins, was characterized in complex with F-actin by cryo-EM reconstruction at 9 Å resolution^13^, and therefore, was selected for this study.

MAS NMR spectroscopy, which is emerging as a mainstream structural biology technique, has been applied to studies into cytoskeleton protein assemblies, such as determination of atomic-resolution structure and dynamics of CAP-Gly domain assembled with polymerized microtubules^33^,^34^, and an investigation of the polymorphism of myelin basic protein associated with actin microfilaments^35^. We have determined the chemical shifts and the secondary structure for the majority of cofilin residues. Using chemical shift perturbation analysis in conjunction with double-REDOR (dREDOR) filtered methods^36^, we have delineated the cofilin residues forming the interfaces with F-actin as well as residues that are affected allosterically. The outstanding spectral resolution enabled characterization of the cofilin/actin interactions with unprecedented level of detail. The study presented here opens doors for atomic-resolution structural characterization of assemblies formed by F-actin with cofilin/ADF family and other ABPs.

## Results And Discussion

### Resonance assignments of cofilin in complex with actin filaments

**Figure 1:**
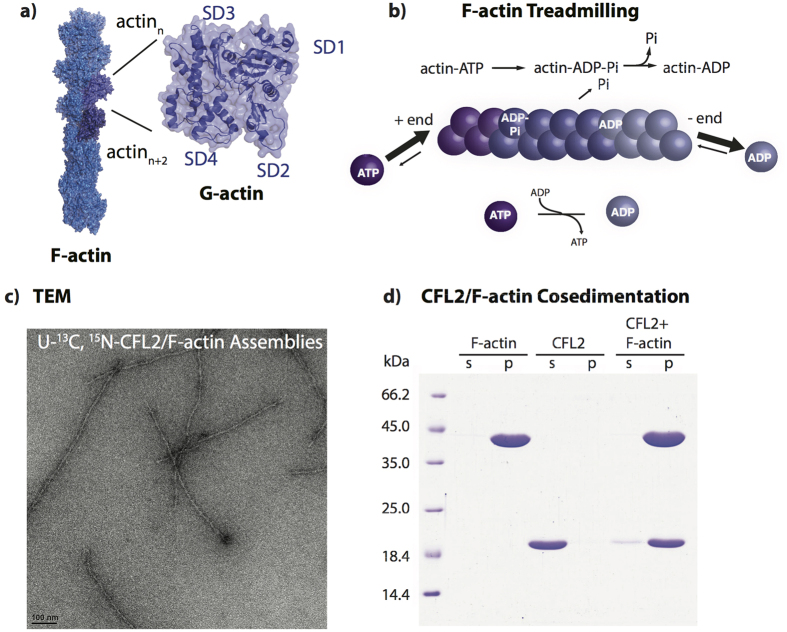
(**a**) F-actin (blue) structure with G-actin protomers (*“n”* and *“n + 2”*) shown in purple. G-actin structure (purple) with subdomains labeled on the structure. (**b**) Actin treadmilling process for the polymerization and depolymerization of actin filaments. (**c**) TEM images of the NMR samples of U-^13^C,^15^N-CFL2 in complex with F-actin. (**d**) SDS-PAGE of CFL2/F-actin co-sedimentation. Samples containing either F-actin (42 kDa), CFL2 (18 kDa), or CFL2 complexed with F-actin were prepared under the conditions replicating the sample preparation for MAS NMR. Following ultracentrifugation, supernatants (s) and pellets (p) were resolved on SDS-gel.

**Figure 2:**
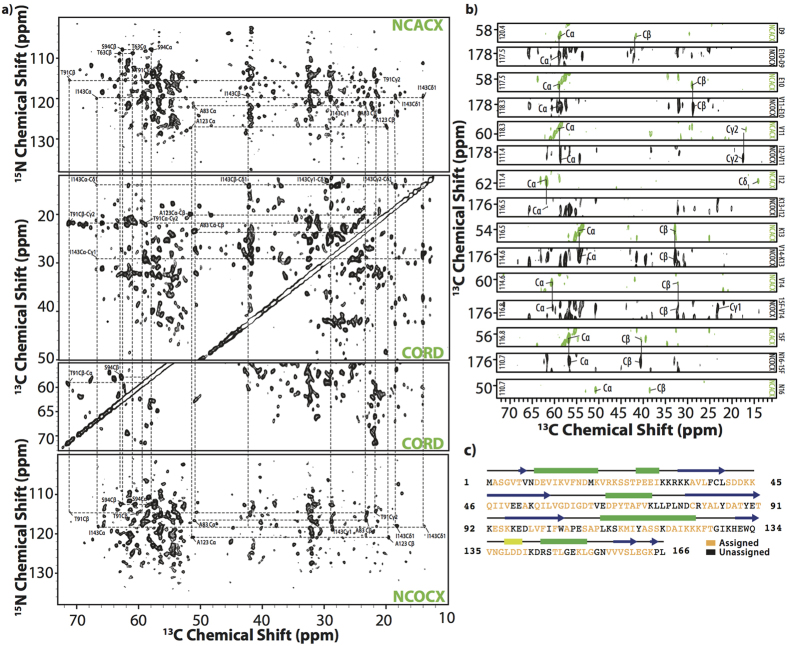
2D and 3D MAS NMR spectra of CFL2 in complex with F-actin, acquired at 19.96 T (^1^H Larmor frequency 850 MHz). (**a**) 2D NCACX (top), CORD (middle), and NCOCX (bottom) spectra. Selected chemical shift assignments and backbone walks are illustrated in the spectra. (**b**) Backbone walk for the stretch of residues D9-N16 obtained from 3D NCACX spectra (green contours) and 3D NCOCX spectra (black contours). (**c**) Human CFL2 sequence with secondary structure of CFL2 determined by TALOS+, where blue arrows represent β-strands, green boxes represent α-helices and yellow box represents 3_10_ helix Colored in yellow and black are residues that are assigned and unassigned (not assigned a single atom), respectively. Spectra were processed with 30 degree sinebell and Lorentizan-to-Gaussian apodization. The first contour is set at 5x the noise level.

The negatively stained TEM image of the NMR sample containing U-^13^C,^15^N human CFL2 in complex with F-actin is shown in Fig. 1c. Formation of CFL2/F-actin complex was confirmed by cosedimentation and SDS-PAGE (Fig. 1d). To corroborate that MAS NMR conditions do not interfere with the sample morphology, TEM images were collected prior to and after the NMR experiments. Spinning of the samples for extended periods of time does not appear to have any effect on the sample morphology. The samples of CFL2/F-actin complexes yield outstanding-resolution MAS NMR spectra, as shown in Fig. 2. For resonance assignments of cofilin in complex with actin filaments, a combination of 2D and 3D homo- and heteronuclear correlation MAS NMR spectra were acquired at 19.96 T using non-uniform sampling (NUS). As shown in Fig. 2a,b and Table S1 (Supplementary Information), remarkably high spectral resolution of 2D and 3D spectra permitted *de novo* resonance assignments of 111 out of 166 residues. Figure 2b displays a backbone walk from D9-N16 using 3D NUS NCOCX and NCACX datasets. The majority of backbone resonances are present in the spectra with the exception of termini residues (M1, P165, L166), several sequence stretches in loop regions spanning residues K30–K34, V72-D79, G130-Q136, and random individual residues throughout the sequence (Fig. 2c and Table S1). These assignments were also corroborated by comparison with solution shifts of free cofilin.

### Intermolecular interface and allosteric changes in cofilin upon binding to F-actin

To determine the intermolecular interface of CFL2 in complex with F-actin, we have pursued two approaches: (i) chemical shift perturbation analysis, and (ii) dREDOR-filter-based experiments allowing for a direct determination of intermolecular dipolar contacts between cofilin and actin filament. This combined analysis yields information on the intermolecular interface and on residues experiencing allosteric changes as the result of the complex formation.

**Figure 3:**
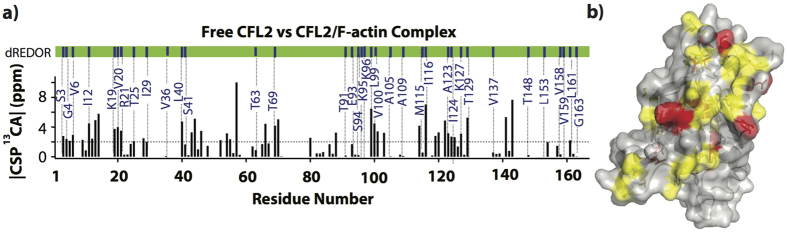
(**a**) C^α^ chemical shift perturbations between free CFL2 and CFL2/F-actin. Residues present in dREDOR experiments of CFL2/F-actin are shown with blue bars above the plot and labeled in blue. Chemical shifts of free CFL2 were determined by solution NMR experiments. Chemical shifts of CFL2/F-actin complexes were determined by MAS NMR experiments. (**b**) The residues constituting the corresponding chemical shift perturbations are mapped onto the CFL1 structure (PDB 1Q8G). Chemical shift perturbations between 2–4 ppm are shown in yellow and chemical shift perturbations above 4 ppm are shown in red.

To probe conformational changes in CFL2 upon binding to F-actin, we have analyzed chemical shift perturbations (CSPs) in free cofilin vs. its complex with F-actin. Since atomic-resolution structure of CFL2 is not yet available, all residues identified in the present study were mapped onto the structure of homologous human CFL1 (PDB 1Q8G) and compared with the cryo-EM reconstruction of CFL2/F-actin complex^13^. The results, summarized in Fig. 3, suggest that the previous assumption that cofilin binds to F-actin as a rigid body are incorrect. Indeed, very large chemical shift perturbations (>2 ppm) are observed for over 27% of all residues, particularly those at the N-terminus (residues S3, G4, V5, T6), in α1-helix (residues D9, V11–V14, K19-R21), β2- and β3-strands (residues L40, Q46, A52, Q54, L55), α3–helix (residues D66, T69, S70), β4- and β5-strands (residues D79, T88, L99-F101, F103), α4–helix (residues K114, I116, S119, S120, D122, A123, I124, K125, K127), the 3_10_ helix (D141), and loop residues (D43, K44, V57, T129, and I142) (Fig. 3). Among these, the α4–helix residues K114, I116, I124, K125, K127 were previously identified to exhibit CSPs upon interaction with G-actin^12^, while most others appear to be specific for binding to F-actin. There are only few residues with strong G-actin induced CSPs^12^ that do not show or show only minor, less than 1 ppm differences, upon binding to F-actin, and most of them are either not conserved between the two cofilins (e.g., A/V137, C/G139 in CFL1/2, respectively) or located nearby the non-conserved residues (e.g., the E134 preceding the non-conserved L/Y135). Since all three residues are located at the actin interface, these changes may contribute to difference in actin-binding properties between CFL1 and CFL2^9^.

**Figure 4:**
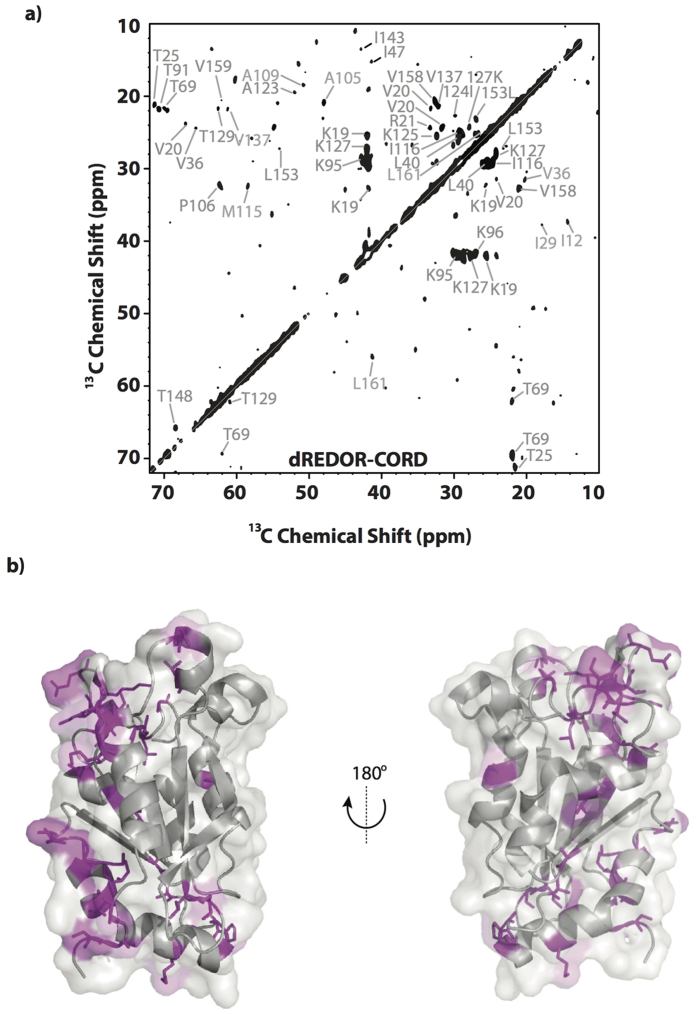
Intermolecular interface of human CFL2 bound to F-actin from MAS NMR. (**a**) dREDOR-CORD spectra for human CFL2 in complex with F-actin. The residues constituting the corresponding intermolecular CFL2/F-actin interfaces were mapped onto the CFL1 structure (PDB 1Q8G) and are shown in purple in (**b**). Spectra were processed with 30 degree sinebell and Lorentizan-to-Gaussian apodization. The first contour is set at 5x the noise level.

The above strong chemical shift perturbations indicate that the corresponding residues either comprise the intermolecular interface with F-actin or undergo allosteric conformational changes upon formation of the complex. To discriminate between those possibilities and identify cofilin residues located at the interface with F-actin, we employed a dREDOR filtered approach^33^. In this method, simultaneous ^1^H-^13^C/^1^H-^15^N REDOR filter dephases all cofilin protons that are directly bonded to either ^13^C or ^15^N atoms (i.e., ^1^H belonging to the U-^13^C, ^15^N-cofilin). The remaining protons, belonging to the interface regions of unlabeled actin, are then used to transfer magnetization to cofilin through ^1^H-^15^N or ^1^H-^13^C cross polarization across the intermolecular interface followed by HETCOR or CORD mixing. The resulting spectrum contains specific information about the residues forming intermolecular interface. We note that dREDOR based experiment is the only approach to identify intermolecular interfaces when chemical shift perturbations are small, such as in cofilin/actin complex here or CAP-Gly/microtubule complex studied by us previously^33^. To perform residue assignments of cofilin residues at the interface, this experiment is implemented as a 2D dREDOR-CORD sequence (Fig. 4a).

### G/F-actin binding sites on cofilin

**Figure 5:**
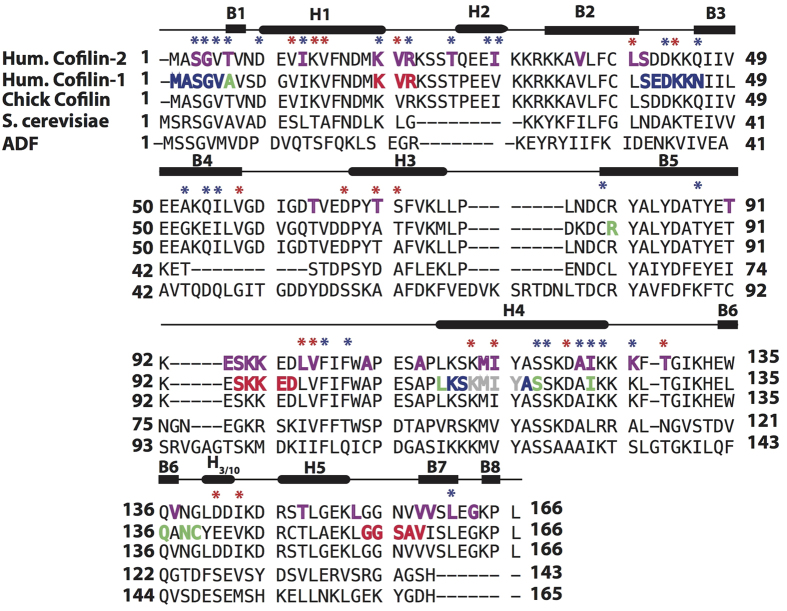
Primary sequences of cofilin/ADF family: human cofilin 2, chick cofilin 2 (PDB 1TVJ), human cofilin 1 (PDB 1Q8G), yeast cofilin (PDB 1COF) and C.elegans actin depolymerizing factor (ADF) (PDB 2MP4). Secondary structure elements are shown above (H denotes α-helices, B – β-strands). Residues previously determined uniquely by solution NMR experiments^12^ and cryo-EM studies^13^ to be involved in G-actin binding are shown on CFL1 sequence in green and blue, respectively. Residues reported to be involved in G-actin binding in both studies are shown in gray. Residues previously determined by cryo-EM experiments^13^ to be involved in the F-actin binding are shown in red. Interface residues determined by dREDOR-based methods are colored in purple on CFL2 sequence. On the primary sequence chemical shift perturbations greater than 2 ppm are indicated by blue asterisk and chemical shift perturbations greater than 4 ppm are indicated by a red asterisk.

The dREDOR-based measurements reveal both the G/F- and F- binding sites on the cofilin surface (representing binding to subunits *“n”* and *“n + 2”* in the filament, respectively) to previously unprecedented level of detail. On the basis of the dREDOR-CORD experiment, we confirmed that the G/F-binding site includes the N-terminus (residues S3, G4 and V6) and the N-terminal half of the bent α4-helix (residues M115 and I116) preceding the kink. Interestingly, the entire actin-binding surface of the α4-helix appears to be shifted towards its C-terminus as compared to that observed in the crystal structure of G-actin with an ADF/cofilin homology domain represented by the C-terminal domain of twinfilin^14^, thus far the only atomic resolution structure of actin-cofilin complex available. Indeed, the N-terminal residues of the α4-helix that constitute the essential part of the G/F-binding site in the C-twinfilin structure (266-IRER-269 corresponding to 111-LKSK-114 in human CFL2) are not present in the dREDOR-CORD spectra. On the other hand, the data indicate that residues A123, I124, K127, T129 at the C-terminal part of the kinked α4-helix, which are not at the G-actin/C-twinfilin interface, are part of the interface in the CFL2/F-actin complex. Residues located in the corresponding part of the α4-helix of budding yeast cofilin were found by mutagenesis to be essential for cofilin function *in vivo*^18^ and were recognized to be affected upon binding to G-actin in synchrotron oxidation experiments^15^ and in solution NMR studies^12^, see Fig. 5. The fact that strong signals corresponding to these residues are found in dREDOR spectra suggests that actin is directly involved in interaction with the terminal part of the α4-helix. It remains to be determined whether the differences observed in the various studies reflect variations in actin-binding modes between the ADF-homology domains of C-twinfilin and cofilin, or between interaction of cofilins with G- vs F-actin, or even between binding of cofilin to ATP- (the X-ray structure) vs ADP-actin (present work). It is also possible that these additional residues contribute to a new F-actin binding site, by being possibly involved in binding to the D-loop of a longitudinally adjacent actin subunit (*“n + 2”*), as it can be speculated based on the proximity of these elements in the cryo-EM reconstruction of F-actin with CFL2^13^. According to the dREDOR results, other residues that might contribute to the G/F-site (i.e. to binding to the *“n + 2”* subunit in the filament) are L40, S41, Q46, A105, A109, V137, N138, which overall is in agreement with previous findings^12^,^14^,^15^,^18^.

### F-actin binding sites on cofilin

**Figure 6:**
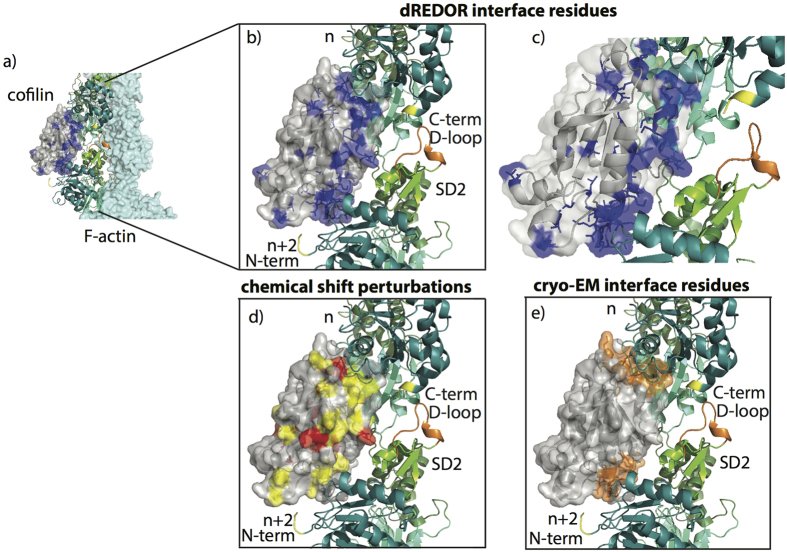
(**a**) Structure of F-actin (cyan) decorated with CFL2 (gray) determined by cryo-EM (PDB 3J0S)^13^. Two adjacent protomers of actin are shown as cartoons. (**b**) CFL2 interface residues S3, G4, V6, I12, K19, V20, R21, T25, I29, V36, L40, S41, T63, T69, T91, E93, S94, K95, K96, L99, V100, A105, A109, M115, I116, A123, I124, K127, T129, V137, T148, L153, V158, V159, L161 and G163 obtained from dREDOR-CORD MAS NMR experiments of CFL2/F-actin are shown in blue. Subdomains of actin protomers (*“n”* and *“n + 2”*) are colored in teal (SD1_*n*_, SD1_*n+2*_), green (SD2), and cyan (SD3, SD4). DNase binding loop (orange), N- and C-termini (yellow) are indicated on the actin structure. (**c**) Zoomed in region of (**b**) of CFL2 interfaces residues obtained from dREDOR-CORD MAS NMR experiments of CFL2/F-actin are shown in blue. (**d**) Chemical shift perturbations, 2–4 ppm (yellow) and above 4 ppm (red), between CFL2/F-actin and free CFL2 mapped onto cofilin structure. (**e**) Interface residues determined from cryo-EM studies mapped onto cofilin structure^13^. Residues M1-V5, K19-R21, S94-D98, K112-S119 and G154-V158 are shown in orange.

To date, the most comprehensive data on the F-site composition originated from a restrained refinement model obtained by fitting atomic-resolution structures of CFL1 (PDB 1Q8G) and actin (PDB 2BTF) to a 9 Å resolution cryo-EM map density of human CFL2 with rabbit skeletal actin^13^. The dREDOR-CORD spectra (Fig. 6) are in excellent agreement with the cryo-EM reconstruction data, and reveal the three major patches of the F-site (to subunit *“n + 2”* in F-actin) (Fig. 6). The first patch is defined by residues 19–21 (also recognized in the cryo-EM study) and 24–26. It is reasonable to speculate that the latter patch can be involved in binding to the N-terminus of actin as these elements are proximal to each other in the cryo-EM reconstruction^13^. The loop comprised by residues 24–32 is present in vertebrates, from fish to human, but not in drosophila, yeast, or plant cofilins. This loop contains a nuclear localization signal (NLS) essential for active translocation of G-actin-cofilin complexes to the nucleus^37^. Involvement of the 24–32 loop in interaction with F-, but not G-actin, suggests a possible mechanism of discriminative recognition of free cofilin and actin-cofilin complexes by nuclear importins. Furthermore, phosphorylation of S23 and/or S24 by PKCα reduces ability of cofilin to bind F-actin and to modify actin dynamics both *in vitro* and *in vivo*^38^. Given a highly acidic nature of actin’s N-terminus, it is reasonable to suggest an electrostatic repulsive mechanism for such inhibition. Therefore, this site represents regulation at the F-actin level, as opposed to the G/F-level regulation via phosphorylation at S3.

The second patch of the F-site is composed of residues 91, 93–96, and 99, 100 (residues 94–98 in the cryo-EM study) within the loop connecting β4-β5 strands and the N-terminal half of β5-strand. The third patch encompasses residues 153, 157–160, and 163 at the C-terminus of cofilin (defined as residues 154–158 by Galkin *et al*.^13^.). It was speculated that overall tighter folding and stronger binding of this regions to F-actin defines the major structural and functional differences between cofilin and ADF^12^. In addition to these well-defined patches, dREDOR spectra revealed residues T63, T69, S70, which are located at the surface opposite to actin binding sites.

As illustrated in Fig. 3, the majority of the residues experiencing large CSPs are either present in the dREDOR spectra or are directly neighboring them corroborating the finding that these residues are located at the interface with F-actin. Among few cofilin residues with large chemical shift perturbations that are not in proximity of residues in dREDOR spectra, V57 (~8 ppm), D141, V143 (4–6 ppm), C80 (~2 ppm), and T88 (>2 ppm), are particularly notable (see Fig. 3). Their unusually large CSPs correlate with localization in loops (V57, C80 and T88) and, therefore, likely reflect flexibility of the corresponding loop regions, or with localization at the actin-cofilin interface (D141, I143) not identified by the dREDOR spectra. Indeed, D141 and D142 of CFL2 correspond to E141 and E142 in CFL1 sequence. This difference was recently implicated as a likely source of different affinities of the two cofilins to ATP-actin^9^. In addition, high CSP of C80 likely reflects changes in the oxidation state of this residue (reduced in free cofilin and oxidized in complex with actin, according to the ^13^C^α^/C^β^ chemical shifts^39^). Additional insights into the nature of the changes described by the large chemical shift perturbations will be gained from the atomic-resolution MAS NMR structure of CFL2 bound to actin, which is forthcoming.

## Conclusions

We reported atomic-resolution structural investigation of the human CFL2 complex with F-actin, by MAS NMR spectroscopy. Using a combination of chemical shift perturbation analysis and dREDOR-based methods, we have determined the cofilin residues comprising the interfaces with F-actin as well as residues affected allosterically. The outstanding spectral resolution enabled characterization of the cofilin/actin interactions with unprecedented level of detail. Broadly, MAS NMR approach reported here is applicable to the analysis of actin-binding proteins bound to filamentous actin that otherwise are not amenable to atomic-resolution studies through other current techniques.

## Methods

### Materials

^15^NH_4_Cl and U-^13^C_6_ glucose were purchased from Cambridge Laboratories, Inc. Common chemicals were purchased from Fisher Scientific or Sigma-Aldrich.

### Expression and Purification of U-^13^C,^15^N-cofilin

Tag-less full-length human CFL2 (cloned between NcoI and BamHI sites in pET15b vector (Novagen)) was expressed in *Escherichia coli* BL21-CodonPlus(DE3) (Agilent Technologies). Transformed bacterial cells were grown at 37 °C in 4 L of rich medium (1.25% tryptone, 2.5% yeast extract, 125 mM NaCl, 0.4% glycerol, 20 mM TRIS-HCl (pH 8.2)) supplemented with 50 μg/mL ampicillin and 34 μg/mL chloramphenicol to OD_600_ 1-1.2. Bacteria were pelleted, washed in MJ medium^40^ without addition of glucose and ammonium chloride, resuspended in 0.75 L of MJ medium without glucose and NH_4_Cl and incubated on a shaker for 1 h at 25 ^°^C. Following this incubation, the bacterial cell suspension was supplemented with U-^13^C_6_ glucose (4 g/L final concentration) and ^15^NH_4_Cl (1 g/L final concentration) and expression was induced with 1 mM IPTG. Cultures were grown overnight at 25 ^°^C. Cells were pelleted at 4 ^°^C, resuspended in ice-cold buffer A (10 mM PIPES, pH 6.8, 0.5 mM EDTA, 10 mM β-mercaptoethanol, 0.5 mM phenylmethylsulfonyl fluoride, 5 mM benzamidine, protease inhibitor cocktail (Sigma)), and lysed using French cell press. Sequential anion and cation exchange chromatography was used to purify isotopically labeled cofilin. Cell lysate was cleared by centrifugation at 40,000 g for 30 min at 4 ^°^C and loaded onto a DE52 (DEAE cellulose, Sigma) column followed by an SP-sepharose (Sigma) column connected sequentially. The columns were disconnected and the protein was eluted from SP-sepharose column with a gradient of 50 to 500 mM NaCl in buffer A. Fractions containing cofilin were combined and further purified using size-exclusion liquid chromatography (SEC FPLC) in a buffer containing 10 mM PIPES, pH 6.8, 25 mM KCl, 1 mM dithiothreitol, 0.4 mM EGTA, 0.1 mM phenylmethylsulfonyl fluoride.

### Preparation of F-actin

Skeletal muscle G-actin was prepared from acetone powder of rabbit skeletal muscle (Pel-Freeze Biologicals) as previously described^41^ and stored in G-buffer (5 mM Tris-HCl, pH 8.0, 0.2 mM CaCl_2_, 0.2 mM ATP, 5 mM β-mercaptoethanol). G-actin was switched from Ca^2+^- to Mg^2+^-bound state by 10-min incubation with 0.1 mM MgCl_2_ and 0.4 mM EGTA and polymerized by addition of buffer containing 20 mM PIPES, pH 6.8, 25 mM KCl, 2 mM MgCl_2_, 5 mM β-mercaptoethanol.

### Preparation of U-^13^C,^15^N-cofilin/F-actin complex

For the preparation of MAS NMR samples, U-^13^C,^15^N-CFL2 and F-actin were mixed at a 1:1.2 molar ratio and centrifuged at 90,000 rpm (RCF 435,400 g) at 4 °C for 1 hour in a TLA 120.2 rotor using a Beckman Coulter Optima MAX-XP ultracentrifuge following overnight incubation on ice. The gel-like pellet was transferred into a 1.9 mm Bruker rotor. 14.3 mg of hydrated U-^13^C,^15^N-cofilin/actin complexes containing an estimated 3 mg of isotopically labeled cofilin were packed into 1.9 mm Bruker rotors.

### Transmission Electron Microscopy

The U-^13^C,^15^N-cofilin/actin sample morphology was verified by transmission electron microscopy (TEM). Actin samples were stained with uranyl acetate (5% w/v), deposited on 400 mesh, formvar/carbon-coated copper grids, and dried. The TEM images were acquired by a Zeiss Libra 120 transmission electron microscope operating at 120 kV.

### Solution NMR Spectroscopy

Solution NMR spectra were acquired on a 14.1 T (^1^H Larmor frequency of 600.1 MHz) Bruker AV spectrometer using a triple-resonance inverse detection (TXI) probe. All spectra of U-^13^C,^15^N-cofilin were recorded at 298 K. Backbone and C^β^ resonance assignments of U-^13^C,^15^N cofilin were carried out using heteronuclear 2D ^1^H-^15^N HSQC and 3D HNCACB, HNCA, HNCO, HNCACO at 298 K.

### MAS NMR Spectroscopy

MAS NMR spectra were acquired on a 19.96 T Bruker AVIII instrument using a 1.9 mm HCN probe. The Larmor frequencies were 850.4 MHz (^1^H), 213.8 MHz (^13^C) and 86.2 MHz (^15^N). The MAS frequency was 14 kHz, controlled to within ±10 Hz by a Bruker MAS controller. The temperature was calibrated using KBr as the temperature sensor. The actual temperature at the sample was 273 K and maintained to within ±0.1 °C using a Bruker temperature controller. ^13^C and ^15^N chemical shifts were referenced with respect to the external standards adamantane and NH_4_Cl, respectively.

Dipolar-based 2D and 3D NCACX and NCOCX experiments were acquired using non-uniform sampling (NUS). The random exponentially weighted NUS schedules were employed, see Supplementary Information^42^,^43^. The 3D spectra were acquired with 25% NUS using 48 complex points in the t_1_ and t_2_ indirect dimensions, with maximum evolution times of 3.4 ms and 6.9 ms for ^13^C and ^15^N, respectively. The spectra were processed using the MINT reconstruction protocol^42^. The signal-to-noise ratio (SNR) of the 2D NCACX and NCOCX were 14 and 13, respectively. The SNR for the 2D ^13^C-^13^C CORD is 15. The first contour level was set to 5X the noise level for all spectra. The uncertainties in the solid-state chemical shifts are ±0.3 ppm. The typical 90° pulse lengths were 2.75 μs, 2.95 μs for ^13^C, and 3.3 μs for ^15^N. The ^1^H-^13^C and ^1^H-^15^N CP employed a linear amplitude ramp for 80–100%: the ^1^H RF field was 91 kHz; and the center of the ramp on the ^13^C or ^15^N was Hartmann-Hahn matched to the first spinning sideband. In 2D and 3D NCACX experiments, the RF field strengths were 64.9 kHz, 84.7 kHz and 91 kHz for ^15^N, ^13^C and ^1^H channels, respectively. The DARR mixing sequence was applied to the ^1^H channel and the DARR mixing time was 50 ms. The ^1^H decoupling powers were 90–100 kHz during acquisition and evolution periods in all experiments.

For 2D ^13^C-^13^C CORD correlation experiments^44^, the typical 90° pulse lengths were 2.55 μs for ^1^H, and 2.3 μs for ^13^C. The ^1^H-^13^C CP employed a tangent amplitude ramp of 80–100%, the ^1^H RF field was 75 kHz, and the center of the ramp of the ^13^C Hartmann-Han matched the first spinning sideband. The RF field on the ^1^H channel was matched to the MAS frequency (14 kHz) and one half of it (7 kHz) during the 50 ms mixing time. The typical decoupling power was 90–100 kHz during the acquisition and evolution. The spectral width was 299.75 ω_2_ and 213.83 in ω_1_ with the carrier frequency set to 96.2 ppm.

The double-REDOR (dREDOR) filtered experiments on U-^13^C,^15^N-CFL2 bound to F-actin employed simultaneous ^1^H-^13^C/^1^H-^15^N REDOR dephasing periods of 714 μs, to eliminate signals from ^1^H (all protons in CFL2) directly bound to ^13^C and ^15^N^33^. The CP contact time was 5 ms and the CORD mixing time was 50 ms. To rule out the possibility that certain signals in the dREDOR based spectra may be artifacts arising from dynamic residues due to insufficient suppression of ^1^H spin polarization, control experiments were conducted as described in our report^45^.

## Additional Information

**How to cite this article:** Yehl, J. *et al*. Structural Analysis of Human Cofilin 2/Filamentous Actin Assemblies: Atomic-Resolution Insights from Magic Angle Spinning NMR Spectroscopy. *Sci. Rep.* **7**, 44506; doi: 10.1038/srep44506 (2017).

**Publisher's note:** Springer Nature remains neutral with regard to jurisdictional claims in published maps and institutional affiliations.

## Supplementary Material

Supplementary Information
