# Supplementary material for: Structural Analysis of Human Cofilin 2/Filamentous Actin Assemblies: Atomic-Resolution Insights from Magic Angle Spinning NMR Spectroscopy
Source: Sci Rep. 2017 Mar 17;7:44506. doi: 10.1038/srep44506 (PMC5355874; doi:10.1038/srep44506)
Supplement: Supplementary Information [file srep44506-s1.pdf]

Supporting Information For

Structural Analysis of Human Cofilin 2/Filamentous Actin Assemblies: Atomic-Resolution Insights from Magic Angle Spinning NMR Spectroscopy

Jenna Yehl[1], Elena Kudryashova[2], Emil Reisler[3,4], Dmitri Kudryashov[2,*] and Tatyana Polenova[1,*]

[1]Department of Chemistry and Biochemistry, University of Delaware, Newark, Delaware 19716, [2]Department of Chemistry and Biochemistry, The Ohio State University, Columbus, OH 43210, [3]Department of Chemistry and Biochemistry, University of California, Los Angeles, CA 90095, [4]Molecular Biology Institute, University of California, Los Angeles, CA 90095

*__Corresponding authors:__ Tatyana Polenova, Department of Chemistry and Biochemistry, University of Delaware, Newark, DE, USA, Tel.: (302) 831-1968; Email: tpolenov@udel.edu; Dmitri Kudryashov, Department of Chemistry and Biochemistry, The Ohio State University, Columbus, OH, USA; Tel.: (614) 292-4848; E-mail: kudryashov.1@osu.edu

Table S1. Summary table of U-^{13}C, ^{15}N-cofilin-2/F-actin resonance assignments by solution and MAS solid-state NMR experiments. Residues present in dREDOR-CORD spectra are shown. The residues found in dREDOR-CORD spectra constitute intermolecular interface with actin.

		Resonance Assignments		Residues Undergoing Conformational Changes Upon Binding to F-Actin
**Residue Number**	**Residue Type**	**Solution NMR Experiments**	**Solid-State NMR Experiments**	**dREDOR-CORD (cofilin/F-actin)**
1	MET			
2	ALA		✓	
3	SER	✓	✓	✓
4	GLY	✓	✓	✓
5	VAL	✓	✓	
6	THR	✓	✓	✓
7	VAL	✓		
8	ASN	✓		
9	ASP	✓	✓	
10	GLU	✓	✓	
11	VAL	✓	✓	
12	ILE	✓	✓	✓
13	LYS	✓	✓	
14	VAL	✓	✓	
15	PHE		✓	
16	ASN		✓	
17	ASP		✓	
18	MET			
19	LYS	✓	✓	✓
20	VAL	✓	✓	✓
21	ARG	✓	✓	✓
22	LYS	✓	✓	
23	SER	✓	✓	
24	SER	✓	✓	
25	THR	✓	✓	✓
26	GLN		✓	
27	GLU		✓	
28	GLU	✓	✓	
29	ILE	✓	✓	✓
30	LYS	✓		
31	LYS			

32	ARG	✓		
33	LYS	✓		
34	LYS	✓		
35	ALA	✓	✓	
36	VAL		✓	✓
37	LEU		✓	
38	PHE			
39	CYS		✓	
40	LEU	✓	✓	✓
41	SER	✓	✓	✓
42	ASP	✓	✓	
43	ASP	✓	✓	
44	LYS	✓	✓	
45	ARG	✓	✓	
46	GLN	✓	✓	
47	ILE		✓	
48	ILE	✓	✓	
49	VAL	✓	✓	
50	GLU	✓		
51	GLU	✓		
52	ALA	✓	✓	
53	LYS	✓		
54	GLN	✓	✓	
55	ILE	✓	✓	
56	LEU	✓	✓	
57	VAL	✓	✓	
58	GLY	✓	✓	
59	ASP		✓	
60	ILE		✓	
61	GLY		✓	
62	ASP	✓	✓	
63	THR	✓	✓	✓
64	VAL	✓		
65	GLU	✓	✓	
66	ASP	✓	✓	
67	PRO	✓	✓	
68	TYR	✓	✓	
69	THR	✓	✓	✓
70	SER	✓	✓	
71	PHE	✓	✓	

72	VAL	✓	✓	
73	LYS	✓		
74	LEU			
75	LEU	✓		
76	PRO			
77	LEU			
78	ASN	✓		
79	ASP	✓		
80	CYS	✓	✓	
81	ARG	✓		
82	TYR	✓	✓	
83	ALA	✓	✓	
84	LEU	✓	✓	
85	TYR	✓		
86	ASP	✓	✓	
87	ALA	✓	✓	
88	THR	✓	✓	
89	TYR	✓		
90	GLU	✓		
91	THR	✓	✓	✓
92	LYS	✓		
93	GLU	✓	✓	✓
94	SER	✓	✓	✓
95	LYS	✓	✓	✓
96	LYS	✓		✓
97	GLU	✓		
98	ASP	✓		
99	LEU	✓	✓	✓
100	VAL	✓	✓	✓
101	PHE	✓	✓	
102	ILE	✓		
103	PHE	✓	✓	
104	TRP	✓		
105	ALA	✓	✓	✓
106	PRO			
107	GLU	✓		
108	SER	✓	✓	
109	ALA	✓	✓	✓
110	PRO			
111	LEU	✓		

112	LYS	✓		
113	SER	✓		
114	LYS	✓	✓	
115	MET	✓	✓	✓
116	ILE	✓	✓	✓
117	TYR	✓		
118	ALA	✓	✓	
119	SER	✓	✓	
120	SER	✓	✓	
121	LYS	✓		
122	ASP	✓	✓	
123	ALA	✓	✓	✓
124	ILE	✓	✓	✓
125	LYS	✓	✓	
126	LYS	✓	✓	
127	LYS	✓	✓	✓
128	PHE	✓	✓	
129	THR	✓	✓	✓
130	GLY	✓		
131	ILE	✓		
132	LYS	✓		
133	HIS	✓		
134	GLU	✓		
135	TRP	✓		
136	GLN	✓		
137	VAL	✓	✓	✓
138	ASN	✓	✓	
139	GLY	✓	✓	
140	LEU	✓	✓	
141	ASP	✓	✓	
142	ASP	✓	✓	
143	ILE	✓	✓	
144	LYS	✓		
145	ASP	✓		
146	ARG	✓		
147	SER	✓		
148	THR	✓	✓	✓
149	LEU	✓	✓	
150	GLY	✓		
151	GLU			

152	LYS		✓	
153	LEU		✓	✓
154	GLY	✓	✓	
155	GLY	✓		
156	ASN	✓		
157	VAL	✓	✓	
158	VAL	✓	✓	✓
159	VAL		✓	✓
160	SER		✓	
161	LEU	✓	✓	✓
162	GLU	✓	✓	
163	GLY	✓	✓	✓
164	LYS		✓	
165	PRO			
166	LEU			

Table S2. Chemical shifts of U-^{13}C,^{15}N cofilin from solution NMR experiments.

		Chemical Shift (ppm)				
Residue		HN	N	Cα	CO	Cβ
1	MET					
2	ALA					
3	SER			57.0	178.2	
4	GLY	8.1	104.3	47.7	174.5	
5	VAL	7.9	120.1	59.1	179.5	29.1
6	THR	7.3	120.2	57.9	177.7	
7	VAL			62.7	175.7	
8	ASN	8.0	123.0	56.0	177.9	
9	ASP	8.2	119.5	56.7	178.8	
10	GLU	8.0	119.0	58.5	179.2	
11	VAL	8.0	120.9	65.0	177.4	
12	ILE	7.7	120.7	64.3	177.7	
13	LYS	7.7	120.7	59.9	178.7	
14	VAL	8.1	118.6	66.4	178.2	31.5
15	PHE					
16	ASN					
17	ASP					
18	MET				177.4	
19	LYS	8.0	117.6	57.9	177.0	31.9
20	VAL	7.2	117.4	62.7	175.7	32.4
21	ARG	8.0	123.0	56.0	176.0	
22	LYS	8.5	123.1	56.8	176.6	33.2
23	SER	8.5	117.3	57.9	174.6	64.1
24	SER	8.6	118.9	59.5		64.4
25	THR	7.1	112.8	61.1		
26	GLN					
27	GLU					
28	GLU			60.0	175.7	
29	ILE	7.6	119.0	66.0	178.4	
30	LYS	7.1	115.5	58.0	177.3	
31	LYS					
32	ARG	8.8	121.6	57.3	176.1	
33	LYS			57.3	175.9	
34	LYS	9.5	124.5	55.1	173.3	
35	ALA	7.8	116.7	51.0	175.5	23.5
36	VAL					
37	LEU					
38	PHE					
39	CYS				172.3	

40	LEU	7.9	118.5	54.2	178.6	
41	SER	9.3	117.6	58.2	176.6	64.3
42	ASP			58.9	177.8	
43	ASP	7.8	120.8	61.9	177.2	33.3
44	LYS			61.9	174.9	
45	ARG	8.7	128.1	55.2	176.2	
46	GLN	9.0	125.7	58.0	178.3	
47	ILE					
48	ILE			62.7	175.8	
49	VAL	8.7	120.1	63.6	175.9	32.4
50	GLU			58.0	178.3	
51	GLU	9.2	120.1	59.3	176.6	
52	ALA	8.7	118.4	53.5	177.8	
53	LYS			57.9	177.3	
54	GLN	8.2	115.6	53.7	174.5	
55	ILE	8.6	119.6	59.8	175.0	
56	LEU	9.4	129.6	53.3	178.2	
57	VAL	9.2	123.7	66.8	179.5	31.2
58	GLY	8.7	105.3	46.0	173.9	
59	ASP					
60	ILE					
61	GLY				173.6	
62	ASP	8.3	120.0	54.6	175.5	47.8
63	THR	7.7	108.0	61.8	174.7	
64	VAL			58.0	177.3	
65	GLU	7.5	117.9	55.4	177.3	
66	ASP	6.6	118.2	52.4	174.6	
67	PRO			64.8	176.2	
68	TYR	8.0	119.9	63.0	177.2	
69	THR	7.8	115.0	66.3	177.5	68.7
70	SER	8.1	116.1	63.5	175.3	62.6
71	PHE	8.0	124.1	60.0	178.6	38.6
72	VAL	8.7	117.1	57.4	177.9	39.9
73	LYS	7.5	117.2	56.1	176.6	41.9
74	LEU					
75	LEU	7.8	120.3	53.2		
76	PRO					
77	LEU					
78	ASN			51.0	173.9	
79	ASP	7.3	114.8	52.8	173.1	43.9
80	CYS	7.6	115.0	57.3	173.9	29.1
81	ARG	9.0	116.3	54.1	175.7	
82	TYR	8.2	115.9	55.1	174.9	41.8

83	ALA	9.5	122.0	51.6	176.2	23.5
84	LEU	8.0	116.2	53.8	174.5	
85	TYR	8.7	122.9	57.1	173.1	
86	ASP	8.0	128.7	52.4	173.3	40.5
87	ALA	8.8	128.5	51.7	177.1	20.3
88	THR	8.4	119.9	62.1	173.2	
89	TYR	9.0	124.4	55.9	172.3	39.2
90	GLU	8.2	116.9	55.0	176.7	
91	THR	8.7	114.4	59.4	174.6	
92	LYS	8.8	117.8	59.0	176.9	
93	GLU	8.0	114.1	57.5	176.4	32.0
94	SER	7.5	112.0	58.2	172.4	66.1
95	LYS	8.3	123.0	56.9	176.0	
96	LYS	8.9	126.4	54.8	173.8	
97	GLU	7.6	117.1	54.7	176.0	32.4
98	ASP	9.0	121.0	54.5	173.6	47.8
99	LEU	8.3	120.0	54.8	176.5	47.8
100	VAL	9.3	123.0	61.4	174.8	35.0
101	PHE	8.7	127.3	56.3	173.9	
102	ILE	9.1	129.8	59.5	174.1	
103	PHE	8.8	126.1	53.4	172.5	39.3
104	TRP	8.9	131.0	55.3	171.8	
105	ALA	7.6	129.3	48.2	172.3	18.0
106	PRO					
107	GLU			58.8	177.3	
108	SER	7.7	108.6	58.2	174.6	63.1
109	ALA	7.3	125.3	50.3	174.4	18.0
110	PRO					
111	LEU	8.8	127.0	58.9	178.8	
112	LYS	8.8	115.7	59.7	179.2	
113	SER	7.1	112.8	61.1	176.1	63.3
114	LYS	8.1	121.2	60.8	179.8	
115	MET	8.5	115.7	59.1	179.4	32.7
116	ILE	7.9	120.9	64.6	179.5	38.0
117	TYR	8.9	121.2	62.1	180.9	
118	ALA	9.0	123.5	56.1	180.5	18.0
119	SER	8.2	119.0	61.2	180.5	63.2
120	SER	7.4	115.0	58.3	172.8	63.7
121	LYS	7.0	121.7	59.7	176.6	31.2
122	ASP	8.4	117.3	57.2	177.4	40.3
123	ALA	7.5	121.2	54.7	179.6	17.6
124	ILE	6.9	114.4	62.9	175.1	
125	LYS	7.7	121.9	59.4	178.9	

126	LYS	7.6	115.6	58.3	178.3	
127	LYS	7.4	115.9	56.0	175.8	32.8
128	PHE	7.6	121.4	56.9	176.1	
129	THR	7.0	111.5	66.1	178.2	
130	GLY	8.1	104.3	47.7	174.5	
131	ILE			61.5	174.5	
132	LYS	8.4	127.8	57.2	175.9	
133	HIS	7.1	116.2	55.4	173.3	34.6
134	GLU	8.9	123.7	54.1	174.7	33.2
135	TRP	9.4	127.7	53.6	173.8	33.3
136	GLN	9.3	129.8	55.2	173.5	
137	VAL	8.5	125.2	60.5	174.5	
138	ASN	9.0	122.9	52.9	174.6	34.3
139	GLY	6.9	104.3	45.1	173.4	
140	LEU					
141	ASP	8.5	114.3	60.0	174.9	
142	ASP	8.9	121.0	58.7	177.6	
143	ILE	8.5	118.4	59.2	178.2	
144	LYS	7.6	118.4	58.5	179.2	
145	ASP			55.2	175.5	
146	ARG	7.9	127.4	59.3	176.6	
147	SER			61.4	175.7	
148	THR	7.9	120.0	65.7		67.9
149	LEU			57.5	173.4	
150	GLY	9.0	122.5	45.1	178.6	
151	GLU					
152	LYS					
153	LEU				177.2	
154	GLY	7.5	103.0	45.9	174.2	
155	GLY	8.7	112.5	46.4	175.0	
156	ASN	8.8	121.9	54.5	175.0	
157	VAL	7.1	110.6	61.7	176.2	
158	VAL	7.5	122.7	63.8		
159	VAL					
160	SER					
161	LEU			54.5	176.2	47.8
162	GLU	10.0	126.9	56.1	176.7	
163	GLY	9.6	105.9	45.2	174.0	
164	LYS					
165	PRO					
166	LEU					

Table S3. Chemical shifts of nitrogen and carbon atoms in U-^{13}C,^{15}N cofilin in complex with F-actin-ADP from MAS NMR experiments.

	Chemical Shift (ppm)										
**Residue**	**N**	**Cα**	**Cβ**	**C'**	**Cγ**	**Cγ1**	**Cγ2**	**Cδ**	**Cδ1**	**Cδ2**	**Cε**
M1											
A2											
S3	120.8	59.8	62.5	176.0							
G4	111.5	45.3									
V5	118.2	60.8	24.7	171.3							
T6	112.5	60.8		178.4	22.0						
V7											
N8											
D9	126.5	59.0	42.0	178.4							
10E	117.9	59.4	29.2	178.4	34.3						
11V	118.1	60.5	31.9	178.1							
12I	111.1	61.8	37.2			28.7			14.2		
13K	116.5	55.0	32.8	176.4	31.0						41.6
14V	114.6	60.6	32.2	175.2		23.3					
15F	116.9	56.7	40.6	176.4							
16N	110.7	50.8	38.6	173.9	173.8						
17D	113.1	60.1	41.9								
18M											
19K	114.4	61.7	32.6	178.0	25.5						
20V	123.5	66.7	31.4	179.4		21.2	19.3				
21R	120.1	52.5	33.6	173.7	24.6			38.9			
22K	120.5	57.1	31.0	175.7							
23S	116.9	58.2	64.0	174.7							
24S	114.0	57.7	62.9	174.3							
25T	114.5	59.0	71.5				22.0				
26Q	116.6	54.0	33.0	172.0	36.4			175.6			
27E	117.2	57.8		174.2	34.7			178.3			
28E	113.6	57.5	28.5	172.4	31.1						
29I	116.9	64.0	37.4	177.4			17.8				
30K											
31K											
32R											
33K											
34K											
35A	115.7	50.9	23.5	175.4							
36V	118.4	65.9	31.3	176.7		23.6	20.4				
37L	117.1	55.5	43.0		25.3				22.4		
38F											

39C		56.7	33.5	180.4							
40L	119.7	58.9	42.0	178.4	29.2				24.5	23.5	
41S	121.4	56.5	61.6	174.6							
42D	118.2	58.7	32.3	178.4							
43D	118.0	58.6	36.9	180.2							
44K	123.4	56.7		178.0	21.8			24.4			38.2
45R	119.4	56.2		173.0				40.5			
46Q	129.5	54.5	30.8	174.5							
47I	129.1	60.4	38.9	173.3		29.8	16.2				
48I	126.7	61.3	34.3	174.0		23.9	19				
49V	121.5	63.6	31.1	175.0		22.4	22.5				
50E											
51E											
52A	115.7	55.8	19.4								
53K											
54Q	114.2	56.9	29.1	175.4							
55I	114.2	62.2	34.4	174.3			21				
56L	126.5	53.8	43.2	175.5	24.1						
57V	123.0		32.5	177.1		25.6					
58G		45.8									
59D	114.7	53.2	43.3	173.9							
60I	120.9	61.6	39.3	176.5		32.8	19.3		13.8		
61G	103.8	45.2		173.3							
62S	126.6	56.0	40.9	178.1							
63T	108.8	60.9	63.3								
64V											
65E		57.1	32.2	177.0	38.0						
66D	116.7	56.9	39.6	176.7							
67P	136.0	63.0	50.7	175.2	27.3						
68Y	112.9	62.0	69.6	175.7	21.9						
69T	111.8	58.6	68.0	171.7							
70S	123.7	59.9	42.3	175.3							
71F	121.3	63.8	32.1	175.8	21.0						
72V	116.1	58.6	32.2	177.1				27.8			42.4
73K											
74L											
75L											
76P											
77L											
78N											
79D											
80C	114.2	54.7	32.2	175.1							
81R											

82Y	116.8	54.7	41.8	175.0						
83A	121.2	51.2	23.6	173.9						
84L	114.0	53.1	41.6	177.9						
85Y										
86D		54.1		172.3						
87A	121.4	51.3	23.6	175.5						
88T	117.7	65.4	68.0				22.8			
89Y										
90E										
91T	115.6	59.6	71.2	174.8			21.8			
92K										
93E	118.9	59.2	29.5	177.0	36.0					
94S	107.8	57.9	67.7							
95K	114.1	56.7		173.5				29.0		42.1
96K										
97E										
98D										
99L	121.6	61.3	34.8		21.1					
100V	118.7	65.9	31.0	178.1		21.7				
101F	130.9	52.9	39.6	173.6						
102I										
103F	128.6	56.6	42.8							
104W										
105A	126.7	48.3	20.2	177.7						
106P				122.4						
107E										
108S		57.9	62.6	174.2						
109A	124.4	50.4	18.3	173.9						
110P	135.7		31.3		26.8			50.2		
111L										
112K										
113S										
114K	118.2	61.6	38.4	177.2	27.0			26.8		
115M	112.4	58.6	32.1	176.4						
116I	112.2	57.6	29.1			25.4				
117Y										
118A	123.3	56.3	18.3	178.2						
119S	109.7	58.4	64.1	176.5						
120S	110.7	61.5	63.2							
121K										
122D	127.2	52.3	40.1	172.8						
123A	127.5	51.5	19.9	176.3						
124I	122.9	65.7	37.6	177.4			17.1		13.1	

125K	120.7	56.8	32.4								
126K	120.9	56.9	32.5	175.4							
127K	121.2	59.1	29.7	175.6	24.4			28.3			
128F	112.4	56.6	32.8	176.1							
129T	112.6	60.8	62.4	175.7			21.9				
130G											
131I											
132K											
133H											
134E											
135W											
136Q											
137V	118.4	61.1	32.4	175.0		21.8					
138N	114.2	53.3	34.4	173.9							
139G	103.8	45.6		176.3							
140L	123.7	57.0	42.4	172.8							
121D	126.2	54.7	41.7	175.9							
142D	124.9	57.9	40.6	178.2							
143I	119.5	59.7	38.5	177.7		26.4	18.9				
144K											
145D											
146R											
147S											
148T	114.1	66.0	68.6	177.7			22				
149L	126.3	60.3	42.0	174.9	32.2						
150G											
151E											
152K		62.6	32.4	176.0				27.4			41.9
153L	117.6	56.7	43.8	178.2	26.9					23.2	
154G	103.9			47.8							
155G											
156N											
157V	119.2	63.2	32.5	175.4		22.5					
158V	119.4	63.5	32.5	175.2		22.3					
159V	121.3	61.5	33.1	176.4		21.3					
160S	115.3	56.9	64.7	171.1							
161L	121.1	56.7	40.9	175.9	27.2						
162E	126.7	55.7	25.9	176.5	34.2						
163G	105.6	45.2		173.9							
164K	120.7	59.8	32.4								
165P											
166L											

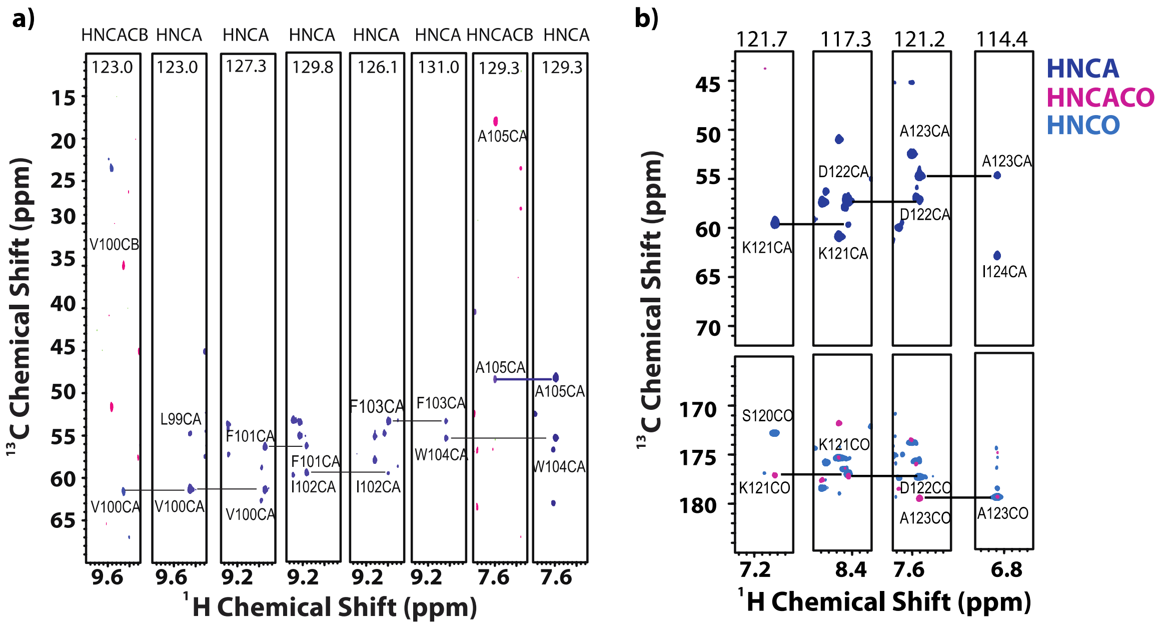

Figure S1. (a) Backbone walk from 3D HNCACB (pink) and HNCA (blue) solution NMR experiments of cofilin-2 for V100-A105 stretch of residues. (b) Backbone walk from 3D HNCACO (pink), HNCA (dark blue) and HNCO (light blue) solution NMR experiments for S120-I124 stretch of residues. The spectra were acquired at 14.1 T (^{1}H Larmor frequency of 600.13 MHz).
